# Characterization of an Integrated Active *Glu-1Ay* Allele in Common Wheat from Wild Emmer and Its Potential Role in Flour Improvement

**DOI:** 10.3390/ijms19040923

**Published:** 2018-03-21

**Authors:** Zhenzhen Wang, Lin Huang, Bihua Wu, Jiliang Hu, Zilong Jiang, Pengfei Qi, Youliang Zheng, Dengcai Liu

**Affiliations:** 1 Triticeae Research Institute, Sichuan Agricultural University, Wenjiang 611130, China; shenhuawzz@163.com (Z.W.); lhuang@sicau.edu.cn(L.H.); hujiliang2017@sina.com (J.H.); zlimba@163.com (Z.J.); pengfeiqi@hotmail.com (P.Q.); ylzheng@sicau.edu.cn (Y.Z.); dcliu7@yahoo.com (D.L.); 2 Key Laboratory of Crop Genetic Resources and Improvement, Ministry of Education, Sichuan Agricultural University, Ya’an 625014, China

**Keywords:** *Triticum turgidum* ssp. *dicoccoides*, *Triticum aestivum*, introgression line, high molecular weight glutenin subunit, *Glu-1Ay*, distant hybridization, flour quality

## Abstract

*Glu-1Ay*, one of six genes encoding a high molecular weight glutenin subunit (HMW-GS), is frequently silenced in hexaploid common wheat. Here, an active allele of *Glu-1Ay* was integrated from wild emmer wheat (*Triticum turgidum* ssp. *dicoccoides*) accession D97 into the common wheat (*Triticum aestivum*) cultivar Chuannong 16 via the repeated self-fertilization of the pentaploid interspecific hybrid, culminating in the selection of a line TaAy7-40 shown to express the wild emmer *Glu-1Ay* allele. The open reading frame of this allele was a 1830 bp long sequence, demonstrated by its heterologous expression in *Escherichia coli* to encode a 608-residue polypeptide. Its nucleotide sequence was 99.2% identical to that of the sequence within the wild emmer parent. The TaAy7-40 introgression line containing the active *Glu-1Ay* allele showed higher protein content, higher sodium dodecyl sulfate (SDS) sedimentation value, higher content of wet gluten in the flour, higher grain weight, and bigger grain size than Chuannong 16. The end-use quality parameters of the TaAy7-40 were superior to those of the medium gluten common wheat cultivars Mianmai 37 and Neimai 9. Thus, the active *Glu-1Ay* allele might be of potential value in breeding programs designed to improve wheat flour quality.

## 1. Introduction

High molecular weight glutenin subunits (HMW-GSs) account for 10% of the total protein deposited in the wheat endosperm and have a major influence over dough quality as a result of their ability to form the aggregate referred to as gluten [1,2,3,4]. The genes encoding the HMW-GS are located at three loci, *Glu-1A*, *Glu-1B*, and *Glu-1D* on the long arms of the homologous chromosomes 1A, 1B, and 1D, respectively [4,5]. It has been confirmed that the two tightly linked genes, *Glu1-1* and *Glu1-2*, at each Glu locus, encode the x- and y-type subunits, respectively. The x-type subunit typically has a higher molecular weight than that of the y-type subunit [6,7]. In spite of the presence of six *Glu-1* genes in common wheat (*Triticum aestivum*, AABBDD, 2*n* = 6*x* = 42) (*1Ax*, *1Dx*, *1Bx*, *1By*, *1Dy*, and *1Ay*), the number of HMW-GS expressed varies from three to five, because the 1Ax and 1By subunits are not usually expressed and 1Ay is always silenced [8,9,10,11] except in two Swedish bread wheat lines [12,13]. The silencing of 1Ay occurs due to either the presence of a premature stop codon [9] or due to the insertion of a transposon-like element within its coding region [14].

Hitherto, several active *Glu-1Ay* genes have been isolated from *Triticum* sources, such as *Triticum timopheevii* ssp. *timopheevii* (AAGG, 2*n* = 4*x* =28) [15], *T. urartu* (AA, 2*n* = 2*x* = 14) [16,17], *T. monococcum* ssp. *aegilopoides* (AA, 2*n* = 2*x* = 14) [18], *T. monococcum* ssp. *monococcum* (AA, 2*n* = 2*x* =14) [16,19], cultivated emmer wheat (*T. turgidum* ssp. *dicoccum*) (AABB, 2*n*= 4*x* =28) [16], and wild emmer wheat (*T. turgidum* ssp. *dicoccoides*) (AABB, 2*n* = 4*x* = 28) [20,21]. It was proposed that the active *Glu-1Ay* genes from the related *Triticum* species could be used for improving wheat processing quality [16,18,22].

Wild emmer wheat, a tetraploid progenitor of common wheat, has wide genotypic variations in agronomic traits, such as yield, grain protein quality and quantity, and resistance to biotic and abiotic stresses [23,24,25,26,27,28,29]. It shares the A and B genomes with common wheat, and introgression is thereby feasible due to the occurrence of homologous recombination between the A and B genomes of wild emmer and common wheat [25,30,31]. Many important traits, such as grain protein content and 1000-kernel weight [32], as well as disease resistance [23,33,34] have been introduced from wild emmer into cultivated common wheat and durum wheat. In contrast, the introgression of storage protein genes from wild emmer wheat has been less reported. A previous study revealed that a *Glu-1Ay* allele derived from wild emmer has the potential to enhance the gluten properties in durum wheat [35]. However, studies on the utilization of wild emmer *Glu-1Ay* allele for common wheat dough quality improvement are rare. Information on the heredity, variation, and expression of the *Glu-1Ay* gene from wild emmer after pentaploid F_1_ hybrid self-crossing eight times is unavailable, and the processing quality effects in common wheat remains unclear.

Our previous study has indicated that wild emmer accession D97 contains active genes at both the *Glu-A1* and the *Glu-B1* loci [29]. In the current study, D97 was crossed with the low-gluten common wheat cultivar “Chuannong 16” (CN16 hereafter) and self-crossing occurred continuously over eight times to introduce an active *Glu-1Ay* into common wheat to enrich the genetic bases at the *Glu-A1* locus. One introgression line TaAy7-40 with desirable agronomic performance was obtained. The objectives of the present study were: (1) to characterize the morphological and cytological attributes of TaAy7-40 and compare them with those of its parents; (2) to isolate, express, and compare the coding sequences of *Glu-1Ay* in TaAy7-40 and its parents; and (3) to study the end-use quality of flour made from TaAy7-40 and evaluate the potential impact of this wild emmer *1Ay* gene on the processing quality of common wheat.

## 2. Results

### 2.1. Phenotype and Karyotype Characteristics

**Figure 1:**
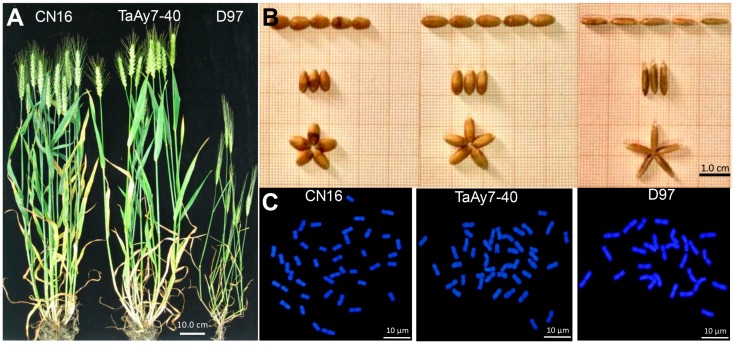
Morphological traits and chromosome patterns of introgression line TaAy7-40 and its parents CN16 and D97. (**A**) plants of TaAy7-40, CN16, and D97; (**B**) seeds of TaAy7-40, CN16, and D97; and (**C**) the number of root-tip chromosomes.

The TaAy7-40 resembled CN16 with respect to plant height, spike, and spikelet number, but all were significantly different from those of wild emmer D97 (Figure 1A, Table 1). Interestingly, the TaAy7-40 had an earlier flowering time than both its parents (Table 1). The grain traits, including kernel length, kernel width, kernel thickness, 1000-kernel weight, and grain weight per plant, showed significant differences between TaAy7-40 and D97, while slight differences occurred between TaAy7-40 and CN16 (Figure 1B, Table 2). Cytological observations confirmed that the chromosome number of TaAy7-40 in root-tip cells was 2*n* = 42 (Figure 1C). Therefore, our results demonstrated that the introgression line TaAy7-40 reached the genetic background of common wheat (AABBDD).

### 2.2. SDS-PAGE Analysis of HMW-GSs

**Figure 2:**
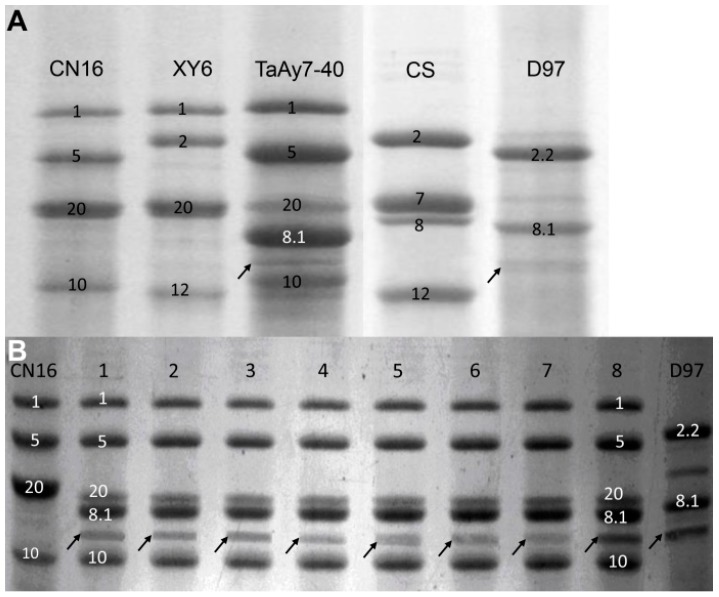
SDS-PAGE analysis of high molecular weight glutenin subunits (HMW-GSs). (**A**) HMW-GSs’ identification of the introgression line TaAy7-40 through the parents and the two common wheat cultivars Xiaoyan6 (XY6) (1Ax1, 1Bx20 + 1By20, 1Dx2 + 1Dy12) and Chinese Spring (CS) (1Bx7 + 1By8, 1Dx2 + 1Dy12) as the references. (**B**) HMW-GSs’ composition and stability of TaAy7-40. Lanes 1–8: eight randomly sampled grains of TaAy7-40.The 1Ay subunits are marked by arrows.

Sodium dodecyl sulfate polyacrylamide gel electrophoresis (SDS-PAGE) analysis showed that the female CN16 had five HMW-GSs, including 1Ax1 at the *Glu-A1* locus, 1Bx20 + 1By20 at the *Glu-B1* locus, and 1Dx5 + 1Dy10 at the *Glu-D1* locus. *1Ay* was not detected in CN16. The male D97 had three HMW-GSs, including 1Ax2.2 [36] + 1Ay at the *Glu-A1* locus and 1By8.1 at the *Glu-B1* locus. However, the resulting introgression line TaAy7-40 possessed six HMW-GSs, including 1Ax1 and 1Ay at the *Glu-A1* locus, 1Bx20 and 1By8.1 at the *Glu-B1* locus, and 1Dx5 and 1Dy10 at the *Glu-D1* locus, compared with the HMW-GSs composition of D97, XY6, and CN16 (Figure 2A). Further analysis by eight randomly sampled grains confirmed that the six HMW-GSs were highly stable in TaAy7-40 (Figure 2B) and that its 1Ay had electrophoretic mobility similar to the fast subunit 1Ay in the D97 profile (Figure 2A,B).

### 2.3. Molecular Characterization of 1Ay Genes

**Figure 3:**
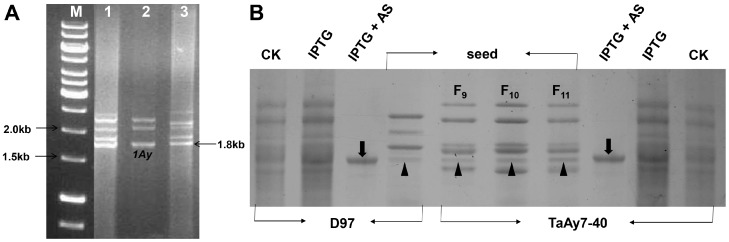
Complete open reading frame (ORF) of HMW-GS amplified by PCR (**A**) and heterologous expression of the *1Ay* gene (**B**). (**A**) PCR amplification of HMW-GS ORFs. Lanes 1, 2, and 3: CN16, D97, and TaAy7-40, respectively. M: DNA marker; the arrowhead indicates the *1Ay* fragment. (**B**) Heterologous expression of the *1Ay* gene of D97 and TaAy7-40 in *Escherichia coli* BL21 (DE3). IPTG, the protein from *E. coli* cultured with isopropyl β-Δ-thiogalactopyranoside (IPTG) using the general extraction method. IPTG+AS, the IPTG induced 1Ay protein extracted using acetone sedimentation (AS). CK indicates the protein from *E. coli* cultured without IPTG using the general traction method. Arrowheads point to the expressed 1Ay subunit protein from *E. coli* culture; triangles point to the 1Ay protein extracted from endosperms of D97 and TaAy7-40 (F_9_ to F_11_ progenies).

**Figure 4:**
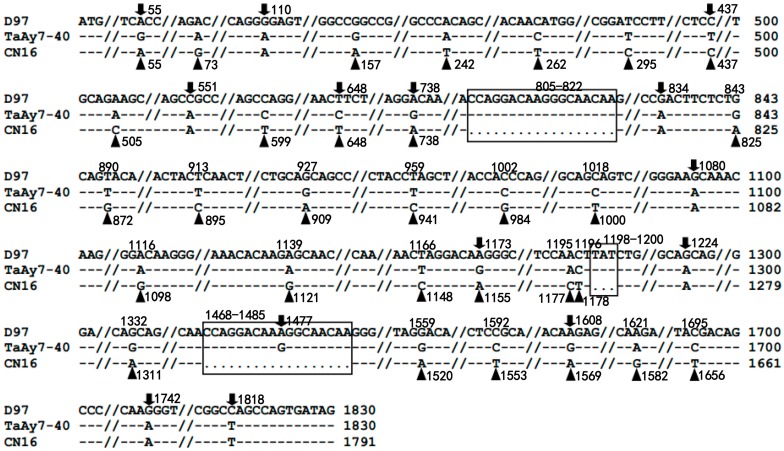
Identification of single nucleotide polymorphisms (SNPs) and insertions and deletions (InDels) of *1Ay* nucleotide sequences in D97 (KC545956), TaAy7-40 (KC545952), and CN16 (KC545955). Triangles mean the different SNPs between CN16 and TaAy7-40; the down arrows indicate the different SNPs between D97 and TaAy7-40; the boxes represent the InDels.

The amplicons consisting of several products were generated from a genomic DNA template of TaAy7-40, CN16, and D97 using the primer pair PF1/PR1 (Figure 3A). Previous studies have demonstrated that the most active *Glu-*1Ay** genes show approximately 1800 bp in length [19,20]. Therefore, in the current study, the PCR bands with such specific fragment sizes were first selected for further cloning and three open reading frames (ORFs) of HMW-GSs sequences were obtained. The *1Ay* ORFs of TaAy7-40 and D97 were each of length 1830 bp and encode for 608 amino acid residues. The *1Ay* ORFs of CN16 were shorter (1791 bp) and a premature stop codon at 1000 to 1002 bp was identified (Figure 4). The authenticity of the *1Ay* alleles isolated from TaAy7-40 and D97 was further confirmed by successful expression in *Escherichia coli* (Figure 3B). The sequences of the three *1Ay* alleles were deposited in GenBank with the accession number KC545952 for TaAy7-40, KC545955 for CN16, and KC545956 for D97.

### 2.4. Sequences Comparison of 1Ay Alleles

In the current study, the sequences isolated from TaAy7-40 (KC545952) and D97 (KC545956) shared a higher level of similarity (from 83.9 to 99.5%) to published *1Ay* ORFs than to either *1By* or *1Dy* ORFs (from 60.7 to 70.7%); their sequences were also quite distinct from any of the genes encoding x-type HMW-GSs, sharing a level of similarity uniformly below 47.0%. The CN16 amplicon sequence (KC545955) was similar (from 77.8 to 98.5%) to that of the *1Ay* ORFs (Table S1). The *1Ay* of D97 had a similarity of 99.2% to the TaAy7-40, whereas both of them shared a relatively lower level of similarity (96.2%, 96.2%) to the *1Ay* sequence of CN16 (Table S1).

Comparison of the three cloned *1Ay* ORFs revealed that a total of three insertions and deletions (InDels) at position 805-822, 1198-1200, and 1468-1485 were detected (Figure 4). Simultaneously, 38 single nucleotide polymorphisms (SNPs), including 32 transitions and six transversions, were identified. In total, 14 SNPs, including 13 transitions and one transversion, were found between D97and TaAy7-40. In contrast, 30 SNPs, including 25 transitions and five transversions, were identified between CN16 and TaAy7-40.

**Figure 5:**
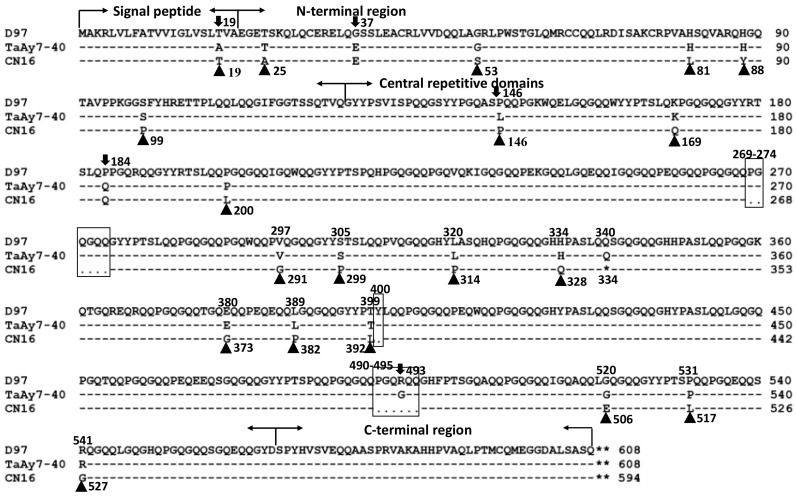
Identification of single amino acid polymorphisms (SAPs) and InDels of the 1Ay amino acid sequences in D97 (KC545956), TaAy7-40 (KC545952), and CN16 (KC545955). Triangles depict the different SAPs between CN16 and TaAy7-40; the down arrows indicate the different SAPs between D97 and TaAy7-40; the boxes represent the InDels. The single star depicts the premature termination codon in CN16 but not in TaAy7-40 and D97.

A similar comparison was made for the predicted polypeptide sequences. All of the three amino acid sequences shared the typical characteristic structure of HMW-GS (Figure 5), which contains a signal peptide, N-terminal, C-terminal, and central repetitive region [2]. Three deletion polymorphisms at position 269–274, 400, and 490–495 were detected in CN16, five single amino acid polymorphisms (SAPs) were identified between TaAy7-40 and D97, and 17 SAPs were found between TaAy7-40 and CN16 (Figure 5). TaAy7-40 and D97 possessed complete 1Ay polypeptide structures, while CN16 encoded a truncated polypeptide as a result of the premature termination codon TAG at position 1000 to 1002 of *1Ay* ORFs.

### 2.5. Phylogenetic Analysis

**Figure 6:**
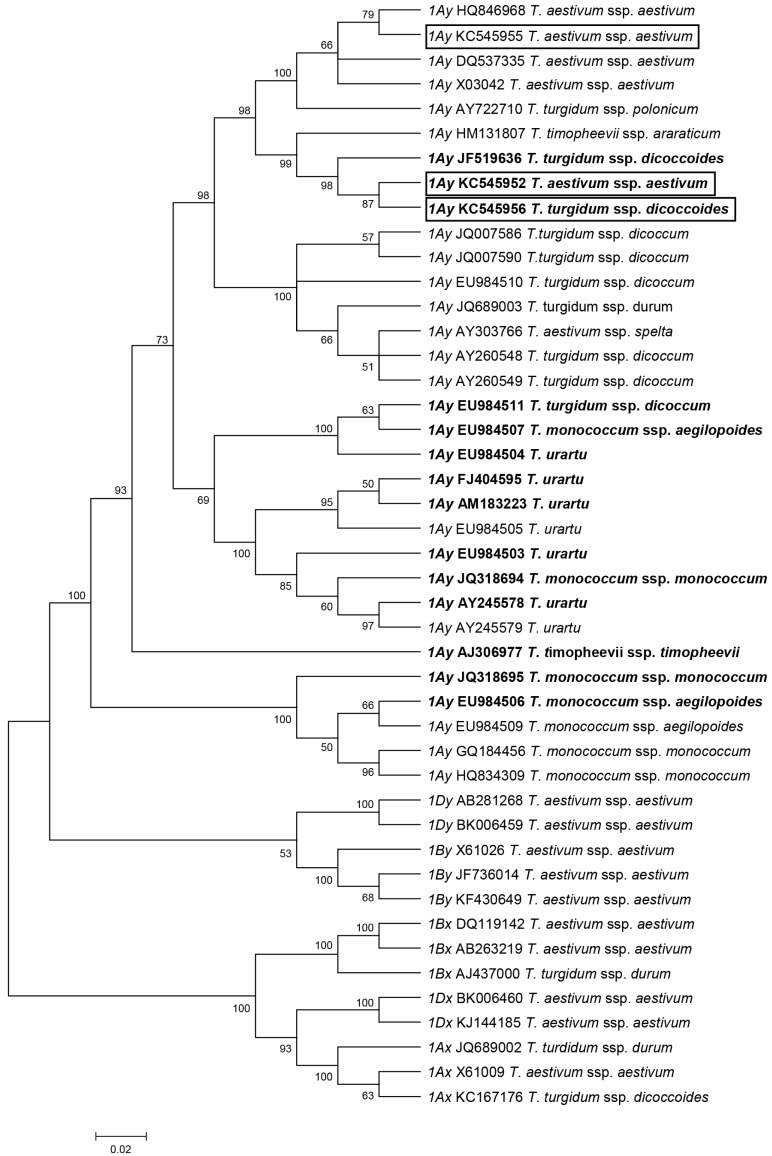
Phylogenetic relationships between the three new *Glu-1Ay* alleles with previously published HMW-GS genes in wheat (*1Ay*, *1By*, *1Bx*, *1Dx*, *1Dy*, *1Ax*). The phylogenetic tree was created based on a multiple alignment of DNA sequences. The three new *1Ay* genes are marked by boxes and the 14 *1Ay* active genes are marked in bold.

A neighbor-joining (NJ) phylogenetic tree was constructed based on the multiple alignment of the full-length sequence of the three cloned *1Ay* alleles, with 29 *1Ay* alleles and 13 other HMW-GS genes that have been previously reported (Figure 6). The result showed that all of the *1Ay* alleles were clustered together and separated from the clades of *1By* and *1Dy* as well as all of the x-type genes, including *Glu-1A*, *Glu-1B*, and *Glu-1D* loci (Figure 6). In the subclade of *1Ay* alleles, the TaAy7-40 *1Ay* (KC545952) and D97 *1Ay* (KC545956) were clustered in a sole clade and clustered closely together with a previously reported active *1Ay* allele in wild emmer (JF519636). In contrast, both of the *1Ay* alleles were estranged from the *1Ay* of CN16 (KC545955).

### 2.6. Processing Quality Parameters (PQPs)

**Figure 7:**
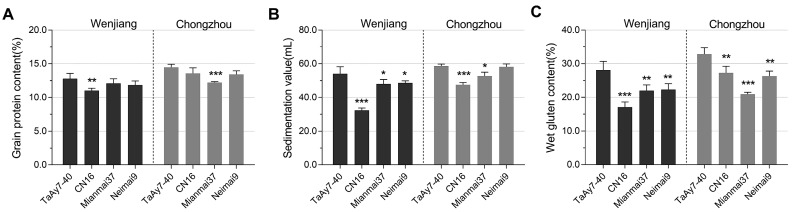
Two-way variance analysis of the difference for three quality traits of introgression lines TaAy7-40, CN16, and the two common wheat cultivars Mianmai 37 and Neimai 9 in Wenjiang and Chongzhou environments. The three PQPs include grain protein content (**A**), sedimentation value (**B**), and wet gluten content (**C**). *, **, and *** represent significance levels at 0.05, 0.01, and 0.001 between TaAy7-40 and each of the other three cultivars, respectively.

Flours milled from TaAy7-40, CN16, Mianmai37, and Neimai9 were compared with respect to their three PQPs: grain protein content, SDS sedimentation value, and wet gluten content. All PQPs scored were significantly higher (*p* < 0.05) for the TaAy7-40 flour than for those of CN16 at two environments except grain protein content at the Chongzhou location (not significant) (Figure 7). Moreover, the grain protein content, SDS sedimentation value, and wet gluten content of TaAy7-40 were also higher than those of the flour milled from the two medium gluten cultivars Mianmai37 and Neimai9. These results indicated that introgression line TaAy7-40 with the traits integrated from wild emmer D97 had better flour processing properties than CN16 and even exceeded the levels of medium gluten cultivars.

## 3. Discussion

The HMW-GS 1Ay is rarely expressed in common wheat. The presence of expressed 1Ay subunits in related *Triticum* species makes it possible to investigate their molecular information and to apply such genes for wheat flour quality improvement. In the present study, we characterized an active *1Ay* gene from wild emmer wheat D97 and transferred this gene into the low-gluten common wheat CN16 via interspecies hybridization. We found that the wild emmer *1Ay* gene can be stably expressed in the common wheat background, and that introgression line TaAy7-40 with six HMW-GSs, including 1Ax1, 1Ay, 1Bx20, 1By8.1, 1Dx5, and 1Dy10, had agronomic properties of common wheat as well as improved processing quality in relation to female CN16.

### 3.1. Identification of Two Novel Active 1Ay Alleles

In the present study, we isolated three *1Ay* sequences from D97 (KC545956), TaAy7-40 (KC545952), and CN16 (KC545955). The KC545955 of CN16 was silenced due to the presence of a premature stop codon in the *1Ay* ORFs (Figure 2 and Figure 5). The authenticity of two functional *1Ay* alleles from wild emmer D97 (KC545956) and the hexaploid introgression line TaAy7-40 (KC545952) was further confirmed by heterogeneous expression in *E. coli* (Figure 3B). The ORFs of KC545956 and KC545952 had identical length (1830 bp) to the majority of *1Ay* sequences from diploid (*T. urartu* FJ404595, EU984503, AM183223, and AY245578) and tetraploid (*T. dococcoides* JF519636, *T. dicoccum* EU984511, and *T. timopheevii* AJ306977) wheat (Table S1). Their deduced proteins shared the typical characteristic structure of HMW-GS, which contains a signal peptide, N-terminal, C-terminal, and central repetitive region (Figure 5) [2]. They were different from the previously published active *1Ay* alleles due to some nucleotide substitutions (Figure S1) and were clustered in a distinct clade from other *1Ay* genes (Figure 6). Therefore, our results demonstrated that the KC545952 and KC545956 sequences identified in our research were two novel *1Ay* alleles.

### 3.2. The 1Ay Sequence Variations

In the current study, the sequence and phylogenetic analyses indicated that the *1Ay* allele of TaAy7-40 (KC545952) shared a high similarity (99.2%) to that of D97 (KC545956). Fourteen SNPs were identified in *1Ay* genes between the TaAy7-40 and D97. A high-fidelity polymerase was used to avoid potential mistakes introduced by PCR amplification, and *1Ay* nucleotide sequences were determined by using sequencing results of multiple single clones. Thus, these sequence variations of *1Ay* alleles observed between TaAy7-40 and D97 are probably not due to errors from the PCR amplification and sequencing.

Wide hybridization has served as an important tool to combine distinct genomes together in the same nucleus [37]. However, as McClintock [38] speculated, this method may create a major “genome shock” that leads to significant genomic restructuring in the hybrid. It has been demonstrated that wide hybridization can produce rich variations in different cross-parents in addition to the parental type [39]. For instance, Yuan et al. [40] reported that wide hybridization of common wheat with rye induces novel DNA variation. Jiang et al. [36] identified four SNPs at the *1Ax2.2* allele between wild emmer and its wide hybrid. It has been demonstrated that repeat sequences are easily changed due to wide hybridization [41] and the large number of similar repetitive sequences may lead to gene duplication and structural chromosomal changes which promote the rapid evolution of genomes [42]. Therefore, the repetitive domain of HMW-GS could be easily affected by the wide hybridization that induced nucleotide variations in the *1Ay* alleles. In addition, the cross-parent genome is asymmetric because strictly speaking, the female common wheat cv. CN16 should have the genome of A_2_A_2_B_2_B_2_DD while wild emmer the genome of A_1_A_1_B_1_B_1_ after a very long evolution, domestication, and genetic improvement history. This genomic asymmetry might drive the genetic variation between *1Ay* alleles of D97 and TaAy7-40, which can lead to purposeful and useful genetic changes [36,43].

### 3.3. A Wild Emmer 1Ay Allele Was Successfully Transferred and Stably Expressed inCommon Wheat

In the comparison between the *Glu-B1* and *Glu-D1* loci, the active alleles at the *Glu-A1* locus in common wheat are of a much lower number because of the silenced versions of *1Ay* [1,11,44,45,46,47]. The transfer of *Glu-D1* and *Glu-A1* loci was successfully carried out in a few previous reports [48,49], and the resulting isohomoeoallelic lines were shown to significantly improve dough and mixing characteristics [50]. Although several active alleles of *1Ay* have been isolated and characterized from wild relatives of wheat, the utilization of these genes for wheat quality improvement has been less-studied. The active 1Ay subunit in two Swedish bread wheat lines had superior quality to subunit 1 or 2* and even higher correlation with a specific Zeleny volume than with the subunits 5+10 [12,13]. Rogers et al. [22] demonstrated that the introgression of *1Ax* and *1Ay* from *T. boeoticum* to the hexaploid common wheat increases gluten strength. However, the grain yield trait of the hybrid hexaploid wheat was not significantly improved. The introgression of the wild emmer *1Ay* locus into durum wheat had a positive effect on enhancing gluten properties [35]. A recent study demonstrated that the expression of an active *1Ay* gene in two Australian wheat cultivars showed a positive effect on wheat quality [51]. To our best knowledge, there are no reports on the introgression of the wild emmer *1Ay* locus into common wheat for the purpose of dough quality improvement.

Wild emmer wheat harbors an abundant HMW-GS composition [19]. Since the *Glu-1Ay* gene is usually silenced in common wheat, the utilization of alien active *1Ay* alleles from wild emmer might be an effective strategy for improving the flour quality of common wheat. However, the genetic improvement of wheat through chromosome engineering done by wide hybridization is more complex than that done via conventional crosses between cultivars of the same species [41]. Introgression of chromatin to wheat from related species often results in deleterious effects on some agronomic traits [52]. It is sometimes possible to recover the original yield by subsequent genetic manipulation, such as backcrossing of lines with alien *Glu* alleles [53,54]. In our previous study, we revealed that the wild emmer HMW-GSs were detected in F_1_ and F_2_ offspring of the interspecies hybridization between common wheat and wild emmer [55]. In the current study, our strategy was to continuously select those single plants with desirable agronomic, high yield, and resistance properties for self-crossing. We identified the novel active *1Ay* allele in an advanced generation hybrid. Introgression line TaAy7-40 turned out to be superior to its progenitor lines D97 and CN16 in most of the tested morphological and grain traits, implying that many other genes from the D97 are present in introgression line TaAy7-40.

We found that the 1Ay subunit with similar electrophoresis mobility to that of the parent wild emmer D97 was detected in introgression line TaAy7-40 after eight times’ self-crossing (Figure 2). Further sequence and phylogenetic analysis revealed that the *1Ay* of TaAy7-40 was highly similar to that of D97 (Figure 6, Table S1). Therefore, our results demonstrated that the active *1Ay* gene from wild emmer could be successfully integrated into common wheat and be stably expressed on a common wheat background. It is believable that wild emmer wheat is beneficial to enriching the genetic bases at the *Glu-A1* locus.

### 3.4. The Wild Emmer 1Ay Might be Partly Associated with Better Flour Quality in Common Wheat

Wheat flour quality is mainly determined by the number and composition of HWM-GSs [3]. That an increasing number of HMW-GS in common wheat might give rise to improvements in dough properties has been confirmed by several studies. For example, Rogers et al. [22] showed that the flour quality of wheat expressing the Glu-A1 subunits 39+40 is superior to that of wheat expressing only the Glu-1Ax1 subunit. Recently, Jiang et al. [41] indicated that the novel HMW-GS 1Ax1.2 or 1Ax2.2 at the *Glu-A1* locus from wild emmer might be responsible for the better dough quality in common wheat. In the current study, the authenticity of the *1Ay* gene from wild emmer was confirmed by heterogeneous expression (Figure 3B) and it was stably expressed in common wheat introgression line TaAy7-40. We demonstrated that TaAy7-40 was significantly superior to its female parent CN16 for not only the SDS sedimentation value and wet gluten content in Chongzhou but also for the three PQPs in Wenjiang (Figure 7) even though they had the same positive effect HMW-GS combination 1Dx5 + 1Dy10 [56]. These differences might to a certain extent have resulted from the various HWW-GSs at the *Glu-1A* locus besides those at the *Glu-1B* locus, because TaAy7-40 had the added novel subunit 1Ay besides the newly substituted subunit 1By8.1 compared with CN16. Previous studies have demonstrated that the expression of active *1Ay* genes has the potential to enhance gluten properties in wheat [12,13,22,35]. Further analyses indicated that TaAy7-40 significantly exceeded the levels of medium gluten cultivars MM37 and N9 in the three PQPs. These results suggested that introgression of the HMW-GSs from wild emmer to common wheat could increase the wheat gluten property in relation to the common wheat acceptor line. That in turn can be co-related to some extent with the active *1Ay*, which is similar to the result by Roy et al. [51] who discovered the expressed 1Ay in Australian wheat cultivars indicates a positive effect on wheat quality.

## 4. Materials and Methods

### 4.1. Plant Materials

The high yielding, low-gluten wheat cultivar CN16 (*T. aestivum*, AABBDD, 2*n* = 6*x* = 42) with the HMW-GSs 1Ax1, 1Bx20 + 1By20, and 1Dx5 + 1Dy10 was crossed as the female parent with the high-protein-content wild emmer accession D97 (*T. turgidum* ssp. *dicoccoides*, AABB, 2*n* = 4*x* = 28, originating from Israel) as the male parent. The resulting pentaploid F_1_ hybrid was advanced to F_9_ (bagging selfing) in order to generate a number of stable 42 chromosome plants expressing desirable agronomic traits. One of these introgression lines, named TaAy7-40, was selected. The end-use quality of flour made from this line was compared to that made from cv.“Mianmai 37” (MM37) and “Neimai 9” (N9), both of which are characterized as medium gluten types. The common wheat cultivars “Xiaoyan 6” (XY6) [57] with the HMW-GSs 1Ax1, 1Bx20+1By20, and 1Dx2+1Dy12 and “Chinese spring” (CS) [58] with null, 1Bx7+1By8, and 1Dx2+1Dy12 were used as standards for the identification of HMW-GSs. All of the materials were maintained at the Triticeae Research Institute, Sichuan Agricultural University, Chengdu, China.

All of the materials for testing processing-quality, including TaAy7-40, CN16, MM37, and N9, were arranged in the field using a randomized complete block design with three replicates at the Wenjiang (30°43′9″ N 103°52′17″ E) and Chongzhou (30°33′34″ N 103°38′35″ E) experimental stations. Individual plants were spaced 10 cm apart within a 2m row, with 30 cm between rows. Each replicate was planted in three rows.

### 4.2. Characterization of Phenotype and Karyotype

In total, 10 morphological traits were studied as listed in Table 1 and Table 2. The plant height, the spike number, and the spikelet number traits were measured in the first culm and spike of each plant. The heading date was recorded at which 50% of the heads had fully emerged from the flag leaf sheath. The flowering time was recorded when anthers were visible on 50% of the plants. The kernel length, width, and thickness were recorded for 30 random kernels from each line, and the average values were used for further statistical analysis. The weight of 300 randomly sampled seeds was recorded with an electronic balance to represent the 1000-kernel weight.

Observations of chromosome number in root-tip cells were performed as described by Zhang et al. [59]. At least 30 root-tip cells were counted for each line. The parent lines D97 and CN16 served as controls.

### 4.3. SDS-PAGE Analysis

The HMW-GSs were extracted from mature grain according to two methods. The first method is the general method for whole protein extraction as described by Wan et al. [60]. The second method is the selective precipitation of the HMW gluten reported by Hu et al. [17]. The HMW-GSs were separated by sodium dodecyl sulfate polyacrylamide gel electrophoresis (SDS-PAGE) according to Hu et al. [17]. To ensure the experimental accuracy and to investigate a HMW glutenin subunit’s stability, at least eight randomly sampled seeds of introgression line TaAy7-40 were analyzed.

### 4.4. DNA Preparation and PCR Amplification

Genomic DNA was extracted from etiolated leaves according to the CTAB method [61] with minor modifications and used as a template to amplify the set of full length *1Ay* genes by priming the reactions via the degenerate pair PF1: 5’-ATGGCTAAGCGGC/TTA/GGTCCTCTTTG-3’, and PR1: 5’-CTATCACTGGCTAA/GGCCGACAATGCG-3’. Since the target sequences are known to have a high GC content, a high-fidelity *ExTaq* polymerase (Takara, Dalian, China) was used for PCR amplifications. The PCR amplifications were performed in a reaction volume of 50 μL containing 5 μL of 10 × *Ex*PCR buffer, 0.2 mmol L^−1^ of dNTPs, 1 μmol L^−1^ of each primer, 2.5 U of *ExTaq* polymerase, and ddH_2_O to 50 μL. PCR was carried out using a Mastercycler pro PCR (Eppendorf, Hamburg, Germany). The cycling program consisted of 94 °C for 5 min, followed by 28 cycles of 94 °C for 40 s, 68 °C for 6 min, and a final extension at 72 °C for 10 min [19]. The amplified PCR products were visualized by 1.0% agarose gel electrophoresis followed by staining with ethidium bromide. The PCR amplification was performed in triplicate for each sample.

### 4.5. Cloning, Sequencing, and Prokaryotic Expression of the ORFs of 1Ay

The amplified products with expected size were recovered, purified, and further ligated into *pMD19-T* vector (TaKaRa, Dalian, China), and the ligation mixtures were transformed into *Escherichia coli* DH10B-competent cells. Three to four sub-clones of these fragments were selected using the nested deletion method [62]. The sequencing was performed by the BGI Company (Shanghai, China) and TaKaRa Biotechnology (TaKaRa). The final nucleotide sequence for each *1Ay* ORF was determined from the sequencing results of three independent clones.

The cloned *1Ay* sequence was re-amplified using the primer pair 6-Ay-F1 (5’-ACCCATATGGAAGGTGAGACCTCTAAGC-3’) and 6-Ay-R1 (5’-TTCCTCGAGCTATCACTGGCTAGCCGAC-3’) to remove the signal peptide and add *Nde I* and *Xho I* restriction sites. The resulting fragment was inserted into an expression vector *pET-30a* (Invitrogen, Waltham, USA) and transformed into *E. coli* BL21 (DE3) plysS cells. The recombinant cells were grown at 37 °C on 2×YT medium containing 25 µg/mL kanamycin and 34 µg/mL chloromycetin until the OD_600_ reached 0.6. The expression of *1Ay* in *E. coli* was induced by the addition of 1mM IPTG for 4–6 h, and the protein output of the cells was extracted as described by Wan et al. [60]. The electrophoretic mobility of the protein expressed in the recombinant cells was compared with that of its native form present in D97 and TaAy7-40 (progenies F_9_ to F_11_).

### 4.6. Sequence Alignmentand Phylogenetic Analysis

The ORFs of HMW-GSs were obtained using the ORF Finder program (www.ncbi.nlm.nih.gov/orffinder). Multiple sequence alignment at both the nucleotide and peptide levels was facilitated by the Clustal X 2.0 software (The Conway Institute, Dublin, Ireland) with manual adjustments where necessary. Sequence alignment of TaAy7-40, D97, and CN16 was performed using MEGA 5.0 [63], and the equivalent analysis at the polypeptide level was done by the GeneDoc2.6 software (genedoc.software.informer.com). The phylogenetic tree was constructed using the neighbor-joining (NJ) algorithm in the MEGA 5.0 software. The bootstrap values in the phylogenetic tree were estimated based on 1000 replications.

### 4.7. Flour Processing Quality Determination

Mature seeds were harvested for determination of the flour processing quality. Seeds of TaAy7-40, CN16, MM37, and N9 were conditioned to 14% moisture [64] and milled using the Chopin CD1 AUTO (Renault, Boulogne-Billancourt, France) [65]. Grain protein content was determined using an Infratec 1241 Grain Analyzer (FOSS A/S, Hillerød, Denmark) following the methods given by Pettersson and Eckersten [66]. SDS sedimentation values were obtained using the procedure as described by Axford et al. [67]. Wet gluten content was determined according to American Association of Cereal Chemists (AACC) method 38-12A [68], and the wet gluten content value in each genotype was recorded at a 14% moisture basis. All of the experiments were repeated three times.

### 4.8. Statistical Analysis

All of the agronomical characters and quality parameters were subjected to analysis of variance, and differences among genotypes were tested using Tukey’s two-way test, which were performed using SPSS version 19.0 software (IBM, Armonk, NY, USA).

## 5. Conclusions

An active *Glu-1Ay* allele derived from wild emmer D97 has been successfully integrated and stably expressed in common wheat CN16 through repeated self-fertilization of the pentaploid interspecific hybrid. The hybrid line TaAy7-40 with active *Glu-1Ay* had agronomic properties of common wheat. It was better than CN16 and the medium gluten common wheat cultivars MM37 and N9 in wheat flour quality. Our findings demonstrated that the expression of active *Glu-1Ay* allele in common wheat may have a positive effect on wheat quality, flour quality, and the potential of wild emmer wheat to be a valuable source for enriching the genetic bases at the *Glu-A1* locus.

## Figures and Tables

**Table 1:** Comparison of morphological characteristics of TaAy7-40 and its parents CN16 and D97.

Line	Plant Height * (cm)	Spike Number *	Spikelet Number *	Heading Date * (day)	Flowering Time * (day)
TaAy7-40	93.5 ± 1.2A	12.2 ± 0.9A	18 ± 1A	140 ± 2A	150 ± 2B
CN16	89.3 ± 6.4A	10 ± 1A	19 ± 1A	143 ± 1A	155 ± 1A
D97	79.6 ± 4.6B	7.5 ± 0.9B	12 ± 0.2B	142 ± 2A	155 ± 2A

Note: The data are presented as the mean ± SD. The capital letters A and B indicate the significant differences at 0.01 level with Turkey’s two-way test between each of two comparison samples. * Sample size, *n* = 30 per replicate, and three biological replicates per variety.

**Table 2:** Comparison of grain traits between the TaAy7-40 and its parents CN16 and D97.

Germplasm	Kernel Length * (mm)	Kernel Width * (mm)	Kernel Thickness * (mm)	1000-Kernel Weight ^§^ (g)	Grain Weight Per Plant * (g)
TaAy7-40	7.1 ± 0.3B	3.2 ± 0.2A	3.0 ± 0.2A	46.6 ± 3.9A	10.6 ± 2.8A
CN16	6.4 ± 0.3C	3.4 ± 0.2A	2.9 ± 0.2A	40.0 ± 4.26B	8.9 ± 3.7A
D97	8.7 ± 0.7A	2.0 ± 0.2B	2.1 ± 0.2B	18.8 ± 3.32C	3.6 ± 1.4B

Note: The data are presented as the mean ± SD. The capital letters A, B, and C indicate the significant differences at 0.01 level with Turkey’s two-way test between each of two comparison samples. * Sample size *n* = 30; ^§^ Sample size *n* = 300.

## Supplementary Materials

The supplementary materials are available online at http://www.mdpi.com/1422-0067/19/4/923/s1. Figure S1. Multiple alignment of the full-length sequences of cloned KC545952 (TaAy7-40) and KC545956 (D97) with 12 published active 1Ay alleles; Table S1. Identities of sequences between the KC545952 and 44 other HMW-GS genes from the *Triticum* species.

## Abbreviations

CTAB	Cetyltrimethyl ammonium bromide
HMW-GS	High molecular weight glutenin subunit
InDel	Insertion and deletion
IPTG	Isopropyl b-D-thiogalactopyranoside
ORF	Open reading frame
PCR	Polymerase chain reaction
PQPs	Processing quality parameters
SAP	Single amino acid polymorphism
SDS-PAGE	Sodium dodecyl sulfate polyacrylamide gel electrophoresis
SNP	Single nucleotide polymorphism
