# Supplementary material for: Characterization of an Integrated Active Glu-1Ay Allele in Common Wheat from Wild Emmer and Its Potential Role in Flour Improvement
Source: Int J Mol Sci. 2018 Mar 21;19(4):923. doi: 10.3390/ijms19040923 (PMC5979310; doi:10.3390/ijms19040923)
Supplement: Supplementary file 1 [file ijms-19-00923-s001.zip › Supplementary/Figuer S1.pdf]

KC545952ATGGCTAAGCGGTTGGTCCTCTTTGCGACAGTAGTCATTGGCCTCGTGTCTCTCGCCGTCGCTGAAGGTGAGACCTCTAAGCAACTACAGTGCGAGCGCGAGCTCCAGGAGAGTTCGCTTGAG 123
KC545956--A--G------------- 123
FJ519636--A--A------------- 123
FJ404595--G-----A-----------------G------G-----------------------------A---C--------- 123
EU984503--G-----A-----------------G------G-----------------------------A---C--------- 123
AM183223--G-----A-----------------G------G-----------------------------A---C--------- 123
AY245578------------C-----------------------------------G-----A-----------------G------G-----------------------------A---C--------- 123
JQ318694--G-----A-----------------G------G-----------------------------A---C--------- 123
EU984504--------------A---------------------------------G-----A-----------------G------G-----------------------------A---C--------- 123
EU984507------------C-----------------------------------G-----A-----------------G------G-----------------------------A---C--------- 123
EU984511--G-----A-----------------G------G-----------------------------A---C--------- 123
AJ306977--G-----A-----------------G------G-----------------------------A---C--------- 123
EU984506--C-------G-----A-----------------G------G-----------------------------A---C--------- 123
JQ318695--C-------G-----A-----------------G------G-----------------------------A---C--------- 123

KC545952GCATGCCGGCTGGTCGTGGACCAACAGTTGGCCGGCCGGCTGCCATGGAGCACGGGGCTCCAGATGCGGTGCTGCCAGCAGCTCCGAGATATTAGTGCCAAGTGTCGCCCCGTCGCCCACAGC 246
KC545956--A---- 246
FJ519636--T---- 246
FJ404595---GT---- 246
EU984503--A----GT---- 246
AM183223---GT---- 246
AY245578---GT---- 246
JQ318694---GT---- 246
EU984504---GT---- 246
EU984507---GT---- 246
EU984511---GT---- 246
AJ306977---GT---- 246
EU984506------------------A----------------T---GT---- 246
JQ318695-----------------------------------T---GT---- 246

KC545952CAAGTCGCAAGACAACATGGGCAAACCGCGGTGCCGCCCAAGGGCGGATCCTTCTACCATCGCGAGACCACGCCACTGCAGCAACTCCAACAAGGAATATTTGGGGGAACATCTTCACAAACA 369
KC545956---------------C--- 369
FJ519636---------------T--- 369
FJ404595---------------T---TC------------------A--- 369
EU984503---------------T---TC------------------A--- 369
AM183223---------------T---TC------------------A--- 369
AY245578---------------T---TC------------------A--- 369
JQ318694---------------T---TC------------------A--- 369
EU984504---------------T--C-- 369
EU984507---------------T--C-- 369
EU984511---------------T--C-- 369
AJ306977---------------T--C-- 369
EU984506---------------T--C---T-- 369
JQ318695---------------T--C---T-- 369

```
KC545952GTACAAGGGTATTACCCAAGTGTAATATCTCCTCAGCAGGGGTCATATTATCCAGGCCAAGCTTCTCTACAACAGCCAGGAAAATGGCAAGAACTAGGACAAGGGCAACAATGGTACTATCCA 492
KC545956-------------------------------------------------------------------C------------------------------------------------------- 492
FJ519636-------------------------------------------------------------------C------------------------------------------------------- 492
FJ404595-------------------------------------------------------------------C----------A--------------------------------G----------- 492
EU984503-------------------------------------------------------------------C-------------------------------------------G----------- 492
AM183223-------------------------------------------------------------------C----------A--------------------------------G----------- 492
AY245578-------------------------------------------------------------------C-------------------------------------------G----------- 492
JQ318694-------------------------------------------------------------------C-------------------------------------------G----------- 492
EU984504-------------------------------------------------------------------C-------------------------------------------G----------- 492
EU984507-------------------------------------------------------------------C-------------------------------------------G----------- 492
EU984511-------------------------------------------------------------------C------------------------G------------------G----------- 492
AJ306977-------------------------------------------------------------------C-------------------A-----------------------G----------- 492
EU984506--------------------C----C-----------------------------------------C--------------------------C----------------G----------- 492
JQ318695--------------------C----C-----------------------------------------C--------------------------C----------------G----------- 492

KC545952ACTTCTCTGCAGAAGCCAGGACAAGGGCAACAAGGGTACTACCGAACTTCTCTGCAGCAGCCAGGACAAAGGCAACAAGGGTACTACCGAACTTCTCTGCAGCAGCCAGGACAAGGGCAACAG 615
KC545956----------------------------------------------------------C---------------------------------------------------------------- 615
FJ519636----------------------------------------------------------A---------------------------------------------------------------- 615
FJ404595------------C---------------------------------------------A----------G----------------------------------------------------- 615
EU984503------------C---------------------------------------------A----------G----------------------------------------------------- 615
AM183223------------C---------------------------------------------A----------G----------------------------------------------------- 615
AY245578------------C---------------------------------------------A----------G----------------------------------------------------- 615
JQ318694------------C---------------------------------------------A----------G----------------------------------------------------- 615
EU984504------------C--.............................................---------G----------------------------------------------------- 570
EU984507------------C--.............................................---------G----------------------------------------------------- 570
EU984511------------C--.............................................---------G----------------------------------------------------- 570
AJ306977------------C--.............................................---------G----------------------------------------------------- 570
EU984506------------C-------------------G...........................A--------G------------------C---------------------------------- 588
JQ318695------------C-------------------G...........................A--------G------------------C---------------------------------- 588

KC545952ATAGGACAATGGCAACAAGGGTACTACCCAACCTCTCCGCAGCACCCAGGACAAGGGCAACAACCAGGACAAGTGCAAAAAATAGGACAAGGGCAACAACCAGAAAAAGGGCAACAACTAGGG 738
KC545956--------------------------------T-----------------------------------------------------------------------------------------A 738
FJ519636--------------------------------T-----------------------------------------------------------------------------------------A 738
FJ404595--------------------------------T-----------------------------------------------------------------------------------------A 738
EU984503--------------------------------T-----------------------------------------------------------------------------------------A 738
AM183223--------------------------------T-----------------------------------------------------------------------------------------A 738
AY245578--------------------------------T-----------------------------------------------------------------------------------------A 738
JQ318694--------------------------------T-----------------------------------------------------------------------------------------A 738
EU984504--------------------------------T-----------------------------------------------------------------------------------------A 693
EU984507--------------------------------T-----------------------------------------------------------------------------------------A 693
EU984511--------------------------------T-----------------------------------------------------------------------------------------A 693
AJ306977--------------------------------T----T-------------------------------------------------------------T----------------------A 693
EU984506--------------------------------T---------------------------------------------C--------------------------C-----G------C---A 711
JQ318695--------------------------------T---------------------------------------------C--------------------------C-----G------C---A 711
```

```
KC545952CAAGAGCAACAAATAGGACAAGGGCAACAACCAGAACAAGGGCAACAACCAGGACAAGGGCAACAACCAGGACAAGGGCAACAAGGGTACTACCCAACTTCTCTGCAGCAGCCAGGACAAGGG 861
KC545956-----------------------------------------------------------------------------------------------G--------------------------- 861
FJ519636-----------------------------------------------------------------------------------------------A--------------------------- 861
FJ404595-----------------------------------------------------------------------------------------------A-------C----------A-------- 861
EU984503-----------------------------------------------------------------------------------------------A-------C----------A-------- 861
AM183223--------------------------------------------------G--------------------------------------------A-------C----------A-------- 861
AY245578-----------------------------------------------------------------------------------------------A-------C----------A-------- 861
JQ318694------------------------------------------------------------------..................-----------A-------C----------A-------- 843
EU984504----------------------------------G-------------------------------..................-----------A------------------A-------- 798
EU984507----------------------------------G-----------G-------------------..................-----------A--C---------------A-------- 798
EU984511----------------------------------G-------------------------------..................-----------A-----------N------A-------- 798
AJ306977------------------------------------------------------------------..................--A-----T--A--------------------------- 798
EU984506----G-------------------------AT--G------A----------A---------------------------------------T--A-------C------T-T---------- 834
JQ318695----G-------------------------AT--G-----------------A---------------------------------------T--A-------C------T-T---------- 834

KC545952CAACAACCAGGACAATGGCAACAACCAGTACAAG...................................................G..................................... 896
KC545956----------------------------------...................................................-..................................... 896
FJ519636----------------------------------...................................................-..................................... 896
FJ404595----------------------------G-----...................................................-..................................... 896
EU984503----------------------------G-----...................................................-..................................... 896
AM183223----------------------------G-----...................................................-..................................... 896
AY245578----------------------------G-----...................................................-..................................... 896
JQ318694----------------------------G-----...................................................-..................................... 878
EU984504----------------------------G-----...................................................-..................................... 833
EU984507----------------------------G-----...................................................-..................................... 833
EU984511----------------------------G-----...................................................-..................................... 833
AJ306977------------------------G---G-----...................................................-..................................... 833
EU984506-------------------------T--G----TGGCAACAACCAGGACAA....................................TGGCAACAACCAGGACAAGGGCAACAACCAGGACAA 921
JQ318695-------------------------T--G-----GGCAACAACCAGGACAAGGGCAACAACCAGGACAAGGGCAACAACCAGGACAATGGCAACAACCAAGACAAGGGCAACAACCAGGACAA 957

KC545952..GCAACAAGGGTACTACTCAACTTCTCTGCAGCAGCCAGTACAAGGGCAACAAGGGCACTACCTAGCTTCTCAGCACCAGCCAGGGCAGGGGCAACAAGGGCACCACCCAGCTTCTCTGCAG1017
KC545956..-------------------------------------------------------------------------------------------------------------------------1017
FJ519636..-------------------------------------------------------------------------------------------------------------------------1017
FJ404595..----------------C---------------------G-----------------------C----------------------------------------------------------1017
EU984503..----A-----------C---------------------G-----------------------C----------------------------------------------------------1017
AM183223..----------------C---------------------G-----------------------C----------------------------------------------------------1017
AY245578..----A-----------C---------------------G-----------------------C----------------------------------------------------------1017
JQ318694..----A-----------C---------------------G-----------------------C---------------------------------------------------------- 999
EU984504..----------------C---------------------G-----------------------C---------------------------------------------------------- 954
EU984507..----------------C---------------------G-----------------------C---------------------------------------------------------- 954
EU984511..----------------C-------------A-------G-----------------------C---------------------------------------------------------- 954
AJ306977..---------A------C-----------------T---G-----------------------C---------------------------------------------------------- 954
EU984506GG----------------C---------------------G---G-------------------C---------------------A-------------A----------------------1044
JQ318695GG----------------C---------------------G-----------------------C---------------------A-------------A----------------------1080
```

```
KC545952CAGTCAGGACAAGGGCAACAAGGGCACCACCCAGCTTCTCTACAGCAGCCAGGACAAGGGAAACAAACAGGACAGCGAGAACAAAGGCAACAACCAGGACAAGGGCAACAAACAGGACAAGAG1140
KC545956--------------------------------------------------------------G------------------------------------------------------------1140
FJ519636--------------------------------------------------------------A------------------------------------------------------------1140
FJ404595-----------------------------------------G-----------------A--A----------------------------------------------------------G-1140
EU984503-----------------------------------------G-----------------A--A----------------------------------------------------------G-1140
AM183223-----------------------------------------G-----------------A--A----------------------------------------------------------G-1140
AY245578-----------------------------------------G-----------------A--A----------------------------------------------------------G-1140
JQ318694-----------------------------------------G-----------------A--A----------------------------------------------------------G-1122
EU984504----------------------A-------T----------G--------------------A----------------------------------------------------------G-1077
EU984507----------------------A-------T----------G--------------------A----------------------------------------------------------G-1077
EU984511----------------------A-------T--C-------G--------------------A----------------------------------------------------------G-1077
AJ306977-----------------------------------------G--------------------A----------------------------------------------------------G-1077
EU984506-----------------------------------------G--A-----------------A-------------------------------------------------------G--G-1167
JQ318695-----------------------------------------G--A-----------------A----------------------------------------------------------G-1203

KC545952CAACAGCCAGAACAAGAGCAACAACTAGGACAGGGGCAACAAGGGTACTATCCAACTTATCTGCAACAGCCAGGACAAGGGCAACAGCCAGAACAATGGCAACAACCAGGACAAGGTCAACAA1263
KC545956--------------------------------A--------------------------------------------------G---------------------------------------1263
FJ519636--------------------------------A--------------------------------------------------A---------------------------------------1263
FJ404595-----A-------------------C------A---------------------------TC---------------------A---------------------------------------1263
EU984503-----A-------------------C------A----------------------------C---------------------A--------------------------------------G1263
AM183223-----A-------------------C------A---------------------------TC---------------------A---------------------------------------1263
AY245578-----A-------------------C------A----G-----------------------C---------------------A--------------------------------------G1263
JQ318694-----A-------------------C------A----------------------------C---------------------A--------------------------------------G1245
EU984504-----A-------------------C------A--------------------------------------------------A----------------------T----------------1200
EU984507-----A-------------------C------A--------------------------------------------------A----------------------T----------------1200
EU984511-----A-------------------C------A--------------------------------------------------A----------------------T----------------1200
AJ306977---------------A---------C------A--------------------------------------------------A---------------------------------------1200
EU984506-----A-------------------C------A-------------------------C--C---------------------A---------------G-----------------------1290
JQ318695-----A-------------------C------A-------------------------C--C---------------------A---------------G-----------------------1326

KC545952GGGCACTACCCAGCTTCTCTGCAGCAGTCAGGACAAGGACAACAAGGGCACTACCCAGCTTCTCTGCAGCAGCTAGGACAAGGACAACCAGGACAAACGCAACAACCAGGACAAGG.......1379
KC545956--------------------------------------------------------------------------------------------------------------------.......1379
FJ519636--------------------------------------------------------------------------------------------------------------------.......1379
FJ404595A-------------------A------------------------------------A----------------------------------------A-----------------.......1379
EU984503A-------------------A----------------------------------------------------C------------------------A-----------------.......1379
AM183223A-------------------A------------------------------------A---------------C------------------------A-----------------.......1379
AY245578A-------------------A----------------------------------------------------C------------------------A-----------------.......1379
JQ318694A-------------------A----------------------------------------------------C------------------------A-----------------.......1361
EU984504-------------------------------------------------------------------------C------------------------A-----------------.......1316
EU984507-------------------------------------------------------------------------C------------------------A-----------------.......1316
EU984511----------------------------------------------------------------C--------C------------------------A-----------------.......1316
AJ306977-------------------------------------------------------------------------C-----------------------T------------------.......1316
EU984506---------------------------C---------------------------------------------C---------------G--------------------------ACAACCA1413
JQ318695---------------------------C---------------------------------------------C---------------G--------------------------ACAACCA1449
```

```
KC545952........GCAACAGCCAGAACAAGA...............GGAACAATCAGGACAAGGGCAACAAGGGTACTATCCAACTTCTCCGCAGCAACCAGGACAAGGGCAACAACCAGGACAAGGG1479
KC545956........------------------...............-------------------------------------------------------------------------------A--1479
FJ519636........------------------...............-------------------------------------------------------------------------------G--1479
FJ404595........------T-----------...............-------C-------------------------------------A---------------------------------G--1479
EU984503........------T-----------...............-------C-------------------------------------A---------------------------------G--1479
AM183223........------T-----------...............-------C-------------------------------------A---------------------------------G--1479
AY245578........------T-----------...............-------C-------------------------C-----------A---------------------------------G--1479
JQ318694........------T-----------...............-------C-------------------------------------A---------------------------------G--1461
EU984504........------T-----------...............-------C-------------------------C-----C--------A------------------------------G--1416
EU984507........------T-----------...............-------C-------------------------C-----C--------A-------------------G----------G--1416
EU984511........------T-----------...............-------C-------------------------C-----C--------A------------------------------G--1416
AJ306977........------------------...............-C-----C-----------------------------------------------------------------------G--1416
EU984506GGACAAAC------A----G-----GACAACCAGGAGAAAC-C-------------------------C-----C-----------A-----G---------------------------G--1536
JQ318695GGACAAAC------A----G-----GACAACCAGGAGAAAC-C-------------------------C-----C-----------A-----G---------------------------G--1572

KC545952CAACAAGGGCACTTCCCAACTTCT............GGACAAGCGCAACAACCAGGACAAGGCCAACAAATAGGACAAGCGCAACAACTAGGACAAGGGCAACAAGGATACTACCCAACTTCT1590
KC545956------------------------............---------------------------------------------------------------------------------------1590
FJ519636------------------------............---------------------------------------------------------------------------------------1590
FJ404595------------------------............---------------------------------------------------------------------------------------1590
EU984503------------------------............-----G--------------------------------------------------------------------------------C1590
AM183223------------------------............---------------------------------------------------------------------------------------1590
AY245578----------------------T-............----------------------------------------------------A----------------------------------1590
JQ318694------------------------............---------------------------------------------------------------------------------------1572
EU984504------------------------............---------------------------------------------------------------------------------------1527
EU984507------------------------............---------------------------------------------------------------------------------------1527
EU984511------------------------............---------------------------------------------------------------------------------------1527
AJ306977------------------------............-------------------------------------------T-------------------------------------------1527
EU984506--------T----G----------CCGCAGCAGCCA----------------------------------C--------T-------------------------------------------1659
JQ318695--------T----G----------CCGCAGCAGCCA----------------------------------C--------T-------------------------------------------1695

KC545952CCGCAGCAGCCAGGACAGGAGCAACAGTCAAGACAAGGGCAACAGTTAGGACAAGGACACCAACCAGGACAAGGGCAACAATCAGGACAAGAGCAACAAGGCTACGACAGCCCATACCATGTT1713
KC545956-----------------A---------------------------------------------------------------------------------------------------------1713
FJ519636-----------------A---------------------------------------------------------------------------------------------------------1713
FJ404595-T---A-----------A------------G--------------------------------------------------------------------------------------------1713
EU984503-T---------------A------------G--------------------------------------------------------------------------------------------1713
AM183223-T---A-----------A------------G--------------------------------------------------------------------------------------------1713
AY245578-T---------------A------------G--------------------------------------------------------------------------------------------1713
JQ318694-T---------------A------------G--------------------------------------------------------------------------------------------1695
EU984504-T---------------A------------G--------------------------------------------------------------------------------------------1650
EU984507-T--------------GA------------G--------------------------------------------------------------------------------------------1650
EU984511-T---------------A------------G--------------------------------------------------------------------------------------------1650
AJ306977-T------A--------A------------G----------------------------------T-------------------------------------------A-------------1650
EU984506-T---------------A-----G------G------------------------------------A------------------------------------T----A-------------1782
JQ318695-T---------------A------------G------------------------------------A------------------------------------T----A-------------1818
```

```
KC545952AGCGTGGAGCAGCAAGCGGCCAGCCCAAAGGTGGCAAAGGCGCACCATCCGGTGGCACAGCTGCCGACAATGTGCCAGATGGAGGGGGGCGACGCATTGTCGGCTAGCCAGTGATAG1830
KC545956----------------------------G---------------------------------------------------------------------------C------------1830
FJ519636----------------------------A---------------------------------------------------------------------------C------------1830
FJ404595----------------------------A---------------------------------------------------------------------------C------------1830
EU984503----------------------------A----------------------------------------------------------------------------------------1830
AM183223----------------------------A--A-------------------------------------------------------------------------------------1830
AY245578----------------------------A---------------------------------------------T-----------------------------C------------1830
JQ318694----------------------------A---------------------------------------------------------------------------C------------1812
EU984504----------------------------A----------------------------------------------------------------------------------------1767
EU984507----------------------------A---------------------------------------------------------------------------C------------1767
EU984511----------------------------A---------------------------------------------------------------------------C------------1767
AJ306977----------------------------A-----------------------C--------------T-------------------------------------------------1767
EU984506-------------T--T-----------A--------------------T-AC-----G---------------------------------------------C------------1899
JQ318695----------------T-----------A---------------G------AC---------------------------------------------------C------------1935
```

Figure S1: Multiple alignment of the full-length sequences of cloned KC545952 (TaAy7-40), KC545956 (D97) , with 12 published active *1Ay* alleles.
Dash line indicates the identical sequences in the *1Ay* alleles. The capital letters indicate the nucleotide polymorphisms detected in the *1Ay* sequences.
