# Supplementary material for: Characterization of an Integrated Active Glu-1Ay Allele in Common Wheat from Wild Emmer and Its Potential Role in Flour Improvement
Source: Int J Mol Sci. 2018 Mar 21;19(4):923. doi: 10.3390/ijms19040923 (PMC5979310; doi:10.3390/ijms19040923)
Supplement: Supplementary file 1 [file ijms-19-00923-s001.zip › Supplementary/Table S1.pdf]

Table S1. Identities of sequences between the KC545952 and 44 other HMW-GS genes from *Triticum* species.

HMW-GS alleles	GenBank accession	Species	Genome	The size of ORF (bp)	Identity(%)		
					KC545952	KC545956	KC545955
1Ay active genes	KC545952	*T. aestivum* ssp. *aestivum*(TaAy7-40)	AABBDD	1830		99.2	96.2
	KC545956	*T. turgidum* ssp. *dicoccoides* (D97)	AABB	1830	99.2		96.2
	JF519636	*T. turgidum* ssp. *dicoccoides*	AABB	1830	99.5	99.5	96.6
	FJ404595	*T. urartu*	AA	1830	97.6	97.6	95.7
	EU984503	*T. urartu*	AA	1830	97.5	97.4	95.6
	AM183223	*T. urartu*	AA	1830	97.3	97.2	95.5
	AY245578	*T. urartu*	AA	1830	97.3	97.3	95.4
	JQ318694	*T. monococcum* ssp. *monococcum*	AA	1812	96.7	96.7	96.6
	EU984504	*T. urartu*	AA	1767	94.5	94.4	94.5
	EU984507	*T. monococcum* ssp. *aegilopoides*	AA	1767	94.2	94.3	94.3
	EU984511	*T. turgidum* ssp. *dicoccum*	AABB	1767	94.2	94.3	94.3
	AJ306977	*T. timopheevii* ssp. *timopheevii*	AAGG	1767	93.8	93.8	93.9
	JQ318695	*T. monococcum* ssp. *monococcum*	AA	1935	85.2	85.3	78.2
	EU984506	*T. monococcum* ssp. *aegilopoides*	AA	1899	86.4	86.5	84.4
1Ay pseudogenes	JQ007586	*T. turgidum* ssp. *dicoccum*	AABB	1830	97.9	98.0	95.9
	JQ007590	*T. turgidum* ssp. *dicoccum*	AABB	1830	97.9	98.0	95.9
	AY260548	*T. turgidum* ssp. *dicoccum*	AABB	1830	97.7	92.0	95.7
	EU984510	*T. turgidum* ssp. *dicoccum*	AABB	1830	97.5	97.5	95.5
	AY722710	*T. turgidum* ssp. *polonicum*	AABB	1812	97.5	97.6	98.3
	AY303766	*T. aestivum* ssp. *spelta*	AABBDD	1830	97.5	97.4	95.5
	DQ537335	*T. aestivum* ssp. *aestivum*	AABBDD	1812	97.7	97.65	98.5
	X03042	*T. aestivum* ssp. *aestivum*	AABBDD	1812	97.7	97.7	98.5
	HQ846968	*T. aestivum* ssp. *aestivum*	AABBDD	1803	96.8	96. 8	97.6
	AY260549	*T. turgidum* ssp. *dicoccum*	AABB	1830	97.2	97.1	95.2
	HM131807	*T. timopheevii* ssp. *araraticum*	AAGG	1866	96.9	96.9	94.5
	JQ689003	*T. turgidum* ssp. *durum*	AABB	1830	97.5	97.4	95.5
	KC545955	*T. aestivum* ssp. *aestivum*(CN16)	AABBDD	1791	96.2	96.2	
	AY245579	*T. urartu*	AA	1920	92.0	92.0	90.1
	EU984505	*T. urartu*	AA	1920	92.8	92.7	90. 9
	GQ184456	*T. monococcum* ssp. *monococcum*	AA	1953	84.3	84.2	78.0
	HQ834309	*T. monococcum* ssp. *monococcum*	AA	1953	83.9	83.9	77.8
	EU984509	*T. monococcum* ssp. *aegilopoides*	AA	1899	86.3	86.36	84.3
1By8	JF736014	*T. aestivum* ssp. *aestivum*	AABBDD	2166	62.2	62.1	63.9
1By9	X61026	*T. aestivum* ssp. *aestivum*	AABBDD	2121	64.3	64.1	67.0
1By18	KF430649	*T. aestivum* ssp. *aestivum*	AABBDD	2166	62.2	62.1	63.9
1Dy10	AB281268	*T. aestivum* ssp. *aestivum*	AABBDD	1950	60.7	60.9	73.1
1Dy12	BK006459	*T. aestivum* ssp. *aestivum*	AABBDD	1977	70.7	70.7	66.9
1Ax1	X61009	*T. aestivum* ssp. *aestivum*	AABBDD	2496	43.5	41.0	36.7

1Ax1	KC167176	*T. turgidum* ssp. *dicoccoides*	AABB	2496	46.6	42.6	37.5
1Ax1	JQ689002	*T. turdidum* ssp. *durum*	AABB	2481	44.7	39.3	37.2
1Bx7	DQ119142	*T. aestivum* ssp. *aestivum*	AABBDD	2388	36.4	36.4	38.8
1Bx17	AB263219	*T. aestivum* ssp. *aestivum*	AABBDD	2244	34.1	34.55	36.3
1Bx20	AJ437000	*T. turgidum* ssp. *durum*	AABB	2391	38.6	39.6	36. 8
1Dx2	BK006460	*T. aestivum* ssp. *aestivum*	AABBDD	2511	37.3	37.3	43.8
1Dx5	KJ144185	*T. aestivum* ssp. *aestivum*	AABBDD	2523	37.7	37.1	38.7
